# Notes and News

**Published:** 1891-05-23

**Authors:** 


### Notes and News.

Collected and Communicated.

The Clapham Maternity Hospital reports 105 in-patients treated in the year ending March 31st, 1891, 287 cases attended at their own homes, 16 monthly nurses and 24 midwifery students trained. This little hospital is officered by lady doctors, and works in connection with rescue homes. Its aims are excellent, and so is the management, for not a single maternal death is reported either amongst in or out patients, and the expenditure for maintenance was only £364.

The thirty-eighth annual report of the Samaritan Society of St. Thomas's Hospital gives a most encouraging account of the Society's work. Over 250 patients have been sent to convalescent homes, the income being sufficient to relieve all the most pressing cases amongst the discharged patients and to increase the investment in the Funds. Special surgical appliances, trusses, and clothing have also been supplied to a great number of patients. A large majority of patients leaving the hospital are in urgent need of new or additional clothing, and therefore this branch of the Society's work requires special support. Could not some of the very many Dorcas Societies which exist throughout the country do something to meet this need?

Earl Amherst presided on the 14th inst. over the annual meeting and half-yearly election of the British Home for Incurables, held at the Cannon Street Hotel. In proposing the adoption of the report, the Chairman said he regretted that the Board had to meet the subscribers with the statement that the year's expenditure had shown an excess of £1,372 over the receipts. Lord Rothschild had just given them 100 guineas, and he trusted many other wealthy persons would follow that example. The number of incurables maintained in the Home at Clapham was 44, while the pensioners numbered 285. Major Dundas seconded the motion, and the report was adopted, and at the conclusion of the formal business the election was proceeded with.

At the annual meeting of the London Homœopathic Hospital, Great Ormond Street, held on Tuesday (Major W. Vaughan Morgan in the chair), the report stated that great success had attended the endeavour to raise £30,000 for the purpose of rebuilding the hospital. Subscriptions to the fund had come in in a most surprising fashion. When it was first decided upon in 1890, £6,000 had at once been subscribed. This had been quickly added to, so that in three months £15,000 had been promised. At the end of 1890 this had increased to £22,000, while the building fund was now £27,000. This wonderfully successful appeal has, however, caused a falling off in the donations of the past year, which, with an increase in the cost of household articles, has resulted in a deficit of about £500, the first time this has occurred for some years. In-patients numbered 791, against 830 in 1889-90 and 711 in 1888-89. Out-patients amounted to 9,763, as against 10,363 and 9,486 in the two previous years.

The Convalescent House at Arbroath, which is the gift of the late Mr. Alexander Duncan, of Rhode Island, U.S.A., and his brother, Mr. John Duncan, was formally opened on May 1st. The house is a neat, bright, and commodious building, erected from plans prepared by Mr. John Duncan's son. It contains accommodation for 18 beds, and large dining, sitting, and other rooms, and is well and comfortably furnished. The offer of the convalescent home was made to the directors of the Arbroath Infirmary, who cordially accepted it on behalf of that institution, and who are undertaking the management. Infirmary patients will be admitted as convalescents free of charge, and other patients on the moderate charge of 7s. weekly.

The annual meeting of the Glasgow Public Dispensary and Samaritan Society was held in the Religious Institution Rooms on the 30th ult., Sir James King, the President, in the chair. Mr. Meldrum, the Secretary, submitted the report of the Directors, which regretted that owing to the condition of the finances of the dispensary, they had been able to supply the medical officers with but few new instruments. The Samaritan Society, which was formed five years ago, continues to form a useful and much appreciated auxiliary of the dispensary. The Chairman, in moving the adoption of the report, said that with a small expenditure of money a large amount of good was being done by the dispensary. The Rev. Dr. Marshal Lang seconded the motion, which was carried.

The quarterly meeting of the Leeds Workpeople's Hospital Fund was held on the 9th inst., Mr. J. W. Jackman presiding. A very satisfactory report was read, showing that the returns during the last three months were upwards of £300 in excess of the amount for the corresponding period last year. This result was not attained without effort, and thanks were due to many friends outside the organisation. The report went on to speak of the cost of advertising subscriptions. The executive recommended the Committee to start a monthly organ of their own, as it was found they could issue 10,000 copies monthly for less than half what they were now paying in advertising. An application had been made to the Committee for an annual grant from the fund on behalf of the Leeds Nurses' Association. The Chairman, in moving the adoption of the report, supported the claims of this Association, and said he thought it should have a share. The report was adopted, but after some discussion the proposal to establish a magazine was negatived.

A meeting of the Board of Management of the Manchester Royal Infirmary was held in the institution on the 27th ult. Mr. E. S. Heywood, who presided, said that there seemed to be an impression abroad that the Board should make public the reasons for their feeling obliged to decline the offer made by Owens College of a site at Stanley Grove, on which to build and maintain a hospital, to contain 150 beds. A statement is now in preparation, which will contain an account of the Board's action. A number of pension regulations, prepared by the House Committee for nurses at the Infirmary, were afterwards adopted. The regulations provided "that any nurse who shall produce evidence of having attained the age of fifty years, and who shall have been not less than twenty-five years continuously in the service of the Manchester Royal Infirmary, and who shall be, in the opinion of the Board of Management, unfit for further public work, and who is also of satisfactory character, shall be entitled to receive a pension of £25 per annum." The insertion of the clause as to character seems almost superfluous. A woman whose character was not "satisfactory" would scarcely remain for twenty-five years in an institution like the Royal Infirmary.

